# Digital Droplet PCR in Hematologic Malignancies: A New Useful Molecular Tool

**DOI:** 10.3390/diagnostics12061305

**Published:** 2022-05-24

**Authors:** Sara Galimberti, Serena Balducci, Francesca Guerrini, Marzia Del Re, Rossella Cacciola

**Affiliations:** 1 Department of Clinical and Experimental Medicine, Section of Hematology, University of Pisa, 56126 Pisa, Italy; sara.galimberti@unipi.it (S.G.); s.balducci3@studenti.unipi.it (S.B.); guerrinifra@libero.it (F.G.); marzia.delre@unipi.it (M.D.R.); 2 Department of Clinical and Experimental Medicine, Section of Hemostasis, University of Catania, 95123 Catania, Italy

**Keywords:** digital PCR, quantitative PCR, multiplexing PCR, MRD, clonality, NGS, point mutations, hematology

## Abstract

Digital droplet PCR (ddPCR) is a recent version of quantitative PCR (QT-PCR), useful for measuring gene expression, doing clonality assays and detecting hot spot mutations. In respect of QT-PCR, ddPCR is more sensitive, does not need any reference curve and can quantify one quarter of samples already defined as “positive but not quantifiable”. In the *IgH* and *TCR* clonality assessment, ddPCR recapitulates the allele-specific oligonucleotide PCR (ASO-PCR), being not adapt for detecting clonal evolution, that, on the contrary, does not represent a pitfall for the next generation sequencing (NGS) technique. Differently from NGS, ddPCR is not able to sequence the whole gene, but it is useful, cheaper, and less time-consuming when hot spot mutations are the targets, such as occurs with *IDH1*, *IDH2*, *NPM1* in acute leukemias or T315I mutation in Philadelphia-positive leukemias or *JAK2* in chronic myeloproliferative neoplasms. Further versions of ddPCR, that combine different primers/probes fluorescences and concentrations, allow measuring up to four targets in the same PCR reaction, sparing material, time, and money. ddPCR is also useful for quantitating *BCR-ABL1* fusion gene, *WT1* expression, donor chimerism, and minimal residual disease, so helping physicians to realize that “patient-tailored therapy” that is the aim of the modern hematology.

## 1. Introduction

Digital Droplet Polymerase Chain Reaction (ddPCR) is a specific, accurate and time-saving technique capables of accurately quantifying gene expression or detecting point mutations applicable in several hematologic disorders, such as leukemias, lymphomas, myeloma, and chronic myeloproliferative neoplasms, and in transplant field. The ddPCR might provide useful informations in prognostic and therapeutic setting.

## 2. Digital PCR: General Features and Applications

**Figure 1:**
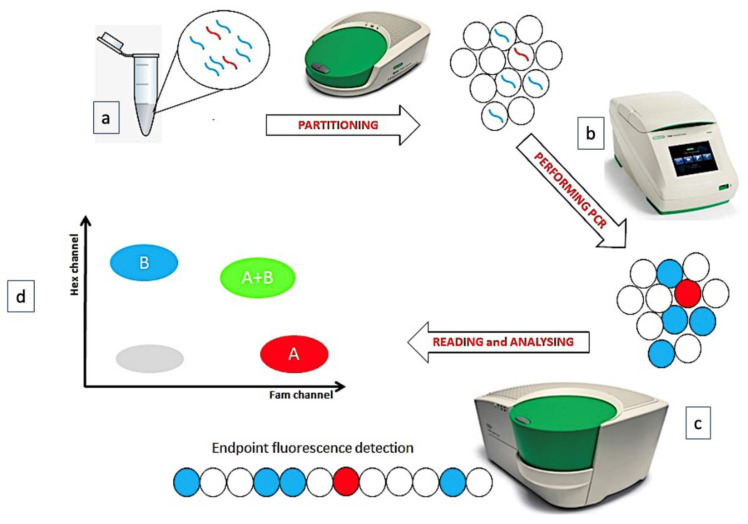
The phases of ddPCR technique. (**a**) The sample is partitioned in many thousands of droplets. (**b**) In each droplet a target is amplified. (**c**) The endpoint amplification results are analyzed. (**d**) A plot is generated, reading 2 fluorescence channels.

Digital Droplet Polymerase Chain Reaction (ddPCR) technique is a recent “version” of quantitative PCR (QT-PCR), based on the partition of sample into several thousand droplets, so that—at least nominally—one single DNA/cDNA copy is partitioned into a single droplet. After the end-point amplification phase, an appropriate software counts and quantifies the numbers of droplets containing the amplified products, applying the Poisson’s correction (it would be possible that any DNA/cDNA molecule enters a droplet or that two or more DNA/cDNA copies would be co-present in a single droplet) (Figure 1) [1].

The first mention of this technique was done by Vogelstein and Kinzler in 1999 [2], but its development effectively occurred after 2012, when instruments with greater than 10,000 partitions per reaction became commercially available, so increasing precision, dynamic range and analytical sensitivity of the new method. Since then, many different applications have been set and published: quantification of donor cell-free DNA in plasma of transplanted patients [3], rapid detection of the most common pathogens in patients with bloodstream infection [4], extracellular RNA [5] or circulating miRNAs quantitation [6].

More recently, ddPCR has been used with success during the Coronavirus pandemics: with a minimum cutoff of 0.04 copies/μL, ddPCR was able to quantify the Coronavirus genome with a sensitivity and specificity of 97.6% and 100%, respectively. Interestingly, in 12 out of 18 patients who converted back to Coronavirus positivity after a negative phase, only ddPCR—and not QT-PCR—still detected viral genome, so reducing the diagnostic error during the recovery phase from the SARS-CoV-2 infection [7]. Moreover, ddPCR accurately quantified Coronavirus genome from crude lysate, with high concordance with measures from purified RNA, thus making more rapid and simpler the viral genome detection [8].

The principal distinctive feature of ddPCR in respect of QT-PCR is that the former does not require a reference standard curve, because the number of “amplified” droplets are divided by the total number of droplets giving an absolute percentage (for example: if 1000 droplets are “positive” among 20,000 total droplets: 1000/20,000 = 0.05%). This is relevant, making possible to quantitate new genes or mutations without need of cloning sequences into “ad hoc” plasmids whose presence in a laboratory significantly increases the probability of environmental contamination. Moreover, the “absolute quantitation” avoids the need of comparing a sample with itself in a different phase of disease, for example before and after a specific treatment, that might overcome the problem of the material consumption.

In terms of sensitivity, ddPCR is at least comparable to QT-PCR, and probably even higher, as shown in several different contexts. In non-Hodgkin’s lymphomas (NHLs) Dr. Drandi and coworkers showed a higher sensitivity for ddPCR (up to one log), especially in samples with very low tumor infiltration [9]. In Waldenstrom’s Macroglobulinemia (WM), it has been shown that ddPCR reached a sensitivity of 5 × 10^−5^, 1.5 log higher than that offered by the Allele-Specific Oligonucleotide PCR (ASO-PCR), the technique classically used for quantitating the rearrangement of the immunoglobulins heavy chain (*IgH*). In a series of 148 patients affected by WM, lymphoplasmacytic lymphoma (LPL) or IgM monoclonal gammopathy of undetermined significance (MGUS), 95% of cases showed the *MYD88L265P* mutation; the concordance with QT-PCR was 74%, and the discordance was always in favor of ddPCR [10]. In patients affected by acute B lymphoblastic leukemia (ALL), ddPCR has been compared to QT-PCR for assessment of minimal residual measurable disease (MRD): rearrangements of *IgH* or of immunoglobulins light chains (*Ig, Ig*), in addition to those of T cell receptors (*TCRs*) have been analyzed and concordant results were observed in 88% of cases, without significant prevalence of one or the two techniques in the discordant cases. On the contrary, 28% of samples defined as “positive but not quantifiable” by QT-PCR resulted quantifiable by ddPCR, so suggesting its higher sensitivity and accuracy [11]. Finally, in multiple myeloma (MM), ddPCR has been shown to have a comparable sensitivity of ASO-PCR in the MRD assessment [12].

**Figure 2:**
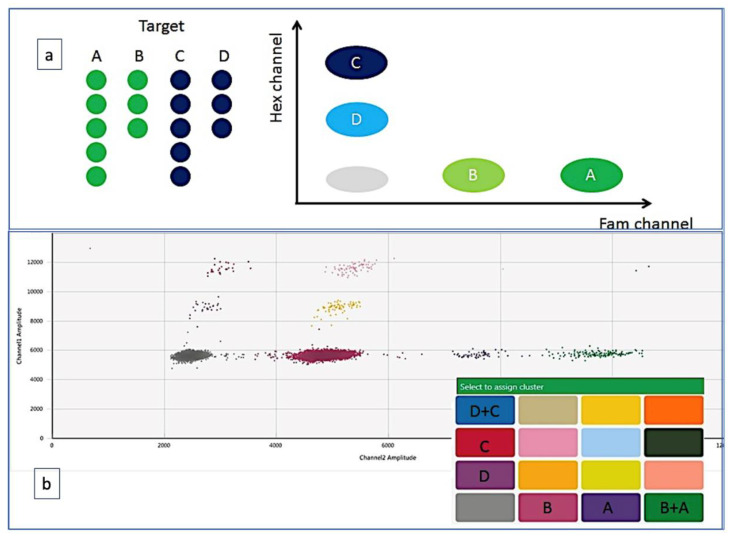
Multiplex assay based on the amplitude of the amplifiers. (**a**) the different targets are detected by probes labeled with the same fluorochrome (FAM or HEX) but used at different concentrations. This strategy allows to quantify four targets within a single reaction (A, B, C, D). (**b**) Targets A and B have relative concentrations of 100% and 50% of FAM-labeled probe, respectively, while C and D have relative concentrations of 100% and 50% of HEX-labeled probe. In the 2D plot, 16 possible clusters are generated: clusters that contain only one target, clusters that simultaneously contain two targets and possibly clusters that contain three targets.

Unfortunately, no guidelines about ddPCR setting are today available; nevertheless, the progressive dissemination of this technique in many laboratories prompted the scientific community to produce two useful documents for producing high-quality assays: the ISO 20395:2019 rules (available at the website https://www.iso.org/obp/ui#iso:std:iso:20395:ed-1:v1:en, accessed on 28 February 2022) and another work that summarized the minimum information for publication of ddPCR experiments (dMIQE guidelines) [13]. In this paper, several technical aspects of ddPCR are discussed: amplicons <150 bp are preferred, the fundamental role of perfectly setting annealing temperature and probe concentrations, the pre-amplification step for low-level targets or the dilution step for too concentrated samples. Finally, the rules for adequately placing the threshold that allows distinguishing positive events from the background and the best number of replicates to do. Moreover, a further application of ddPCR also includes the multiplexing ddPCR [13]. In this case, different fluorescent probes are simultaneously detected in different channels or, in the “higher order multiplexing” version, it is possible to evaluate different targets by varying the concentrations of different probes using the same fluorophore. This most recent version of ddPCR can be also performed by combination of more than one fluorophore with different probe concentrations, to detect up to 4 targets within a single reaction [1,14,15] (Figure 2). During the recent pandemics, the multiplexing ddPCR has been used to simultaneously detect the Coronavirus envelope, the viral RNA polymerase and the nucleocapsid genes, so avoiding the possible mismatch of primers and probes which could follow the virus changes that might lead to false negative results [16].

In the non-invasive prenatal testing field, multiplexing ddPCR using universal locked nucleic acid probes correctly identified several fetal aneuploidies [17], while in oncology this technique detected 4 different PIK3CA mutations on “liquid biopsy”, with a clinical impact in the management of metastatic breast cancer [18]. Moreover, an Italian group set a multiplexing ddPCR in patients affected by chronic myeloid leukemia (CML) with unusual *BCR-ABL1* atypical transcripts, not quantifiable by standardized QT-PCR, with an optimal detection limit level (0.001%). The output of this technique is relevant, because it allows physicians to offer discontinuation of therapy with tyrosine kinase inhibitors even in cases with persistent deep molecular response whose atypical transcripts are difficult to be measured [19].

At the 2022 national meeting of the Italian Society of Experimental Hematology, our group presented a new kind of “higher order multiplexing ddPCR” able to simultaneously measure the expression of *BMI1*, *EZH2*, *USP22* and *GAPDH* genes in 56 patients affected by aggressive B-cell lymphoma (DLBCL). This assay allowed us to analyze very small RNA quantities (the samples were paraffin-embedded and already employed for the cell of origin definition by the Nanostring technology) [20,21].

In conclusion, ddPCR, in its different versions, represents a new, widely applicable, specific, sensitive, and accurate quantitative technique. In the following manuscript, detailed uses of ddPCR in different hematological fields are described. 

## 3. ddPCR Applications in Hematology

### 3.1. ddPCR in Acute Myeloid Leukemia and Myelodysplasias

Acute myeloid leukemia (AML) represents the prototype of a disease where the “target-therapy” is fundamental for improving patients’ survival [22]. In the recent years, the availability of the anti-CD33 monoclonal antibody gemtuzumab ozogamicin [23], of the FLT3 inhibitors (midostaurin and gilteritinib) [24,25] and of IDH1 and IDH2 inhibitors (ivosidenib and enasidenib) [26,27] significantly changed the therapeutic scenario. The increased probability of therapeutic success and the more defined disease genetic features prompted physicians to revise the WHO classification in 2016 [28] and to better define different prognostic classes (at low-, intermediate-, and poor-risk), with consequent different risk-adapted treatment strategies (chemotherapy only for low-risk patients, transplantation for high-risk cases and for those at intermediate-risk but still MRD-positive after the consolidation phase) [29]. Obviously, also the role of the MRD monitoring became more relevant and better standardized [30].

The acute promyelocytic leukemia (APL) was the first AML subtype where ddPCR played a relevant role: indeed, the positive clinical impact of restarting therapy at the re-appearance of the molecular transcript instead of at the hematological relapse is well known [31]. Consequently, the monitoring of *PML-RAR* transcript by a very sensitive technique represents a real clinical need. Recently, two Chinese groups set two new ddPCR assays: the first one measured in the same reaction both *PML-RARα* and *ABL1*, with a higher sensitivity in respect of QT-PCR [32], and the second one published another ddPCR method able to identify at the same time two types of transcripts: with a Limit of Detection (LOD) of 1 × 10^−5^, ddPCR recovered the *PML-RARα* fusion gene in 4% of patients already defined as negative by QT-PCR (whose LOD reached 1 × 10^−4^) [33].

Another ddPCR has been set for detection of *PML-A216V* mutation, already known to be responsible for the resistance to arsenic trioxide. Using ddPCR, 5/13 cases were recognized as mutated versus only 3 by Sanger sequencing; in addition, ddPCR anticipated the mutation appearance by 24, 3 and 4 months compared to Sanger sequencing [34].

In other AML types, ddPCR has been used at diagnosis for distinguishing between two “not otherwise specified” (NOS) forms: the expression levels of the *ANXA3* and *S100A9* genes were increased, whereas those of *WT1* were decreased in the AML-M2 (according to the previous FAB classification) in respect of AML-M1. Moreover, *STMN1* and *ABL1* were upregulated in AMLs with *FLT3* mutations, while *CAT* was over-expressed in the *FLT3*-wild-type cases [35].

In another work, ddPCR for *RUNX1-RUNX1T1* rearrangement was used for MRD assessment in children affected by AML with t(8;21) and compared with flow cytometry and QT-PCR: the flow cytometry lost MRD in 21% of samples (positive by QT-PCR), and ddPCR resulted superior to QT-PCR in one quarter of cases. Finally, in 8 patients with disease progression, the fusion gene was detected by ddPCR but not by QT-PCR before hematological relapse [36].

Another ddPCR assay has been set for detecting and quantitating the *NPM1* mutations, including the rarer ones. Indeed, according to the WHO classification [28], *NPM1* mutations characterize a specific form of AML, and represent the most informative marker of MRD [29,37,38]. Approximately 95% of *NPM1* mutations are represented by nucleotide insertions in exon 12, the most common being type A 75% of cases, and types B and D 15% of the mutated patients. The remaining 10% of patients have rare mutations not covered by the commercially available PCR kits; in this subgroup, ddPCR was able to detect all *NPM1* mutation types, with a sensitivity of 1 × 10^−4^/5 × 10^−5^ [39], a positive predictive power of 100% and a negative predictive power of 94.5% [40]. ddPCR for *NMP1* mutations was also applied to the allogeneic transplantation (alloSCT) setting: with a sensitivity of 0.01% (one log higher than QT-PCR), this technique identified as MRD-positive 33% of patients just before receiving graft. The PCR positivity was the only prognostic factor significantly associated with higher probability of relapse and death [41].

In the complex genomic scenario characterizing AML, ddPCR was also used to track *DNMT3A, IDH1* and *IDH2* mutations: indeed, one third of patients resulted positive for at least one mutation (*DNMT3A > IDH2 > IDH1*), even in hematological complete remission (CR). Moreover, among relapsing patients, 78% resulted ddPCR positive 60 days before, while 75% of patients who remained disease-free were persistently unmutated [42].

**Figure 3:**
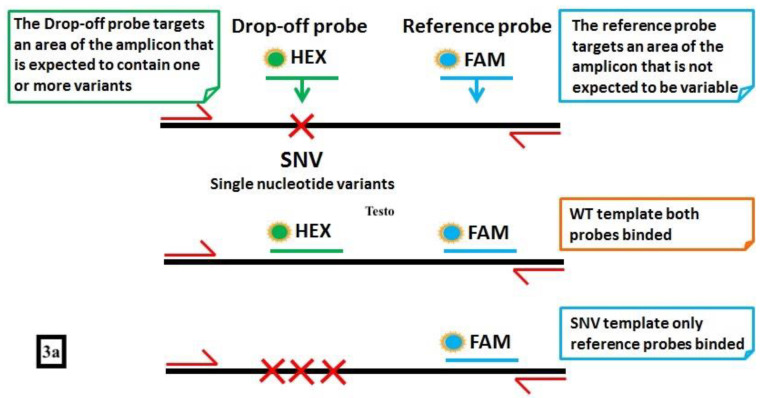

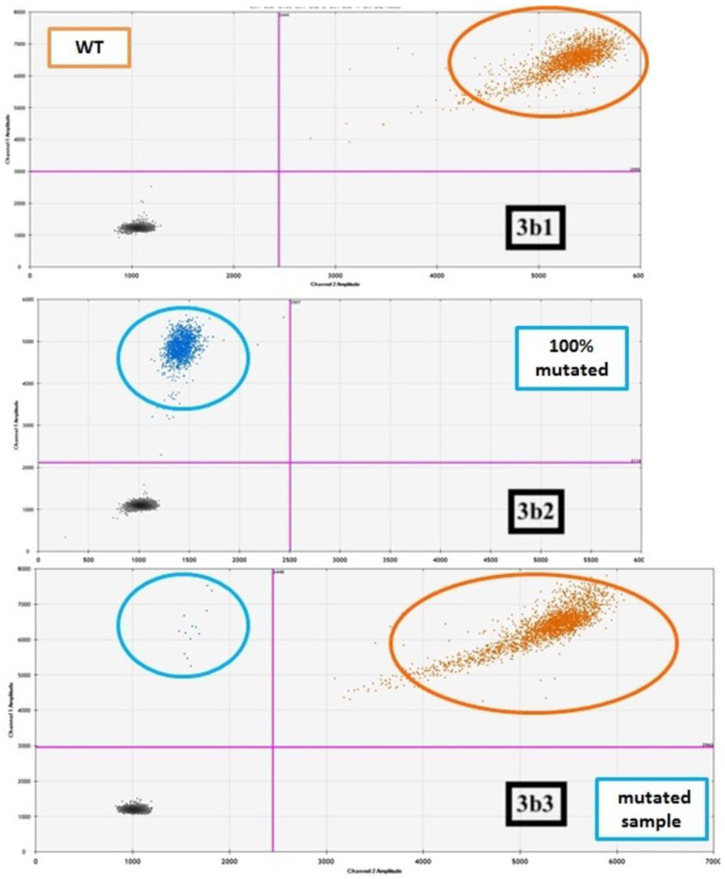
An example of “drop-off” ddPCR (FAM/HEX Assay for *IDH2* mutation). (**a**) This technique requires a single pair of probes to detect and quantify different mutations in a single reaction: the FAM-labeled probe binds a reference sequence distant from the target but within the same amplicon, while the HEX probe binds the wild-type sequence in the target site. Thus, wild-type samples present signals from both FAM and HEX probes, while the mutated ones display only the FAM signal. (**b**) In the 2D plot, samples with different IDH2 genotypes are represented, with channel 1 fluorescence (reference probe) plotted against channel 2 fluorescence (wild-type probe). The droplets are arranged according to the fluorescence levels. In (**b1**), a wild-type (WT) sample represented by a “double positive” population (in orange; reference and wild-type probe in the same droplet) (ref + wt). In (**b2**), a 100% IDH2-mutated case, where only the reference probe (blue = ref) matched with the IDH2 sequence. In (**b3**), a sample carrying the mutation in heterozygosity. This panel represents two droplets’ populations, the (with few events) mutated one (blue) (ref) and the (with a higher number of events) double positive (orange) (ref + wt) one.

*IDH1* and *IDH2* have been reported to be frequently mutated in AML, Myelodysplastic Syndromes (MDS) and chronic Myeloproliferative Neoplasms (MPNs), the most frequent mutations being single-nucleotide variants involving the exon 4 at the arginine hotspot R140 or R172. Many efforts have been done to develop oral *IDH1* and *IDH2* inhibitors, with conflicting results as a monotherapy [43,44], and more promising results in combination with demethylating agents or intensive chemotherapy [45,46]. Being molecular targets for oral therapies, *IDH1/2* have been investigated as markers of MRD, with consistent results when a high-sensibility technique such as ddPCR was used [47], also in post-transplantation setting [42]. A simple and time-sparing method called “drop-off ddPCR” (a multiplexing ddPCR) has been recently described and validated by our group for the simultaneous detection of the *IDH2* most common mutations in codon 140 (Figure 3). With this technique, 60 AML patients at diagnosis have been screened for *IDH2* mutations by the Sanger sequencing, amplification refractory mutation system (ARMS) PCR or ddPCR. With one log more of sensitivity, ddPCR and ARMS PCR identified *IDH2* mutations in 21.6% of cases vs 13.5% of Sanger. Interestingly, ddPCR allowed to identify one of the 4 possible mutations in a single reaction versus the 4 needed reactions for the ARMS PCR. When *IDH2* mutations have been monitored during follow-up, they predicted the hematological relapse in two third of cases, so making *IDH2* attracting even as MRD marker [48]. *c-KIT* activating point mutations have been described in solid tumors, but they seem to have a relevant role both in AML, particularly in CFB-AML [49], and in the systemic mastocytosis [50]. Thus, ddPCR has been applied for detection of *c-KIT* mutations in AML, being associated with a higher relapse rate and poorer outcome [51,52].

If the importance of ddPCR as a diagnostic tool is well-recognized, in the recent years it has been used also for MRD assessment: Petterson et al. monitored MRD in 14 AML patients and were able to produce information about clonal/sub-clonal evolution during treatment and different disease phases [53]. Among the possible MRD markers, ddPCR has been applied for the detection of the Wilm’s Tumor 1 (*WT1*) gene [54]. In a series of 49 AML patients already in deep molecular response by QT-PCR, ddPCR was able to distinguish a subgroup with the best prognosis [55].

A further field of ddPCR application in myeloid malignancies includes the possibility of measuring methylated DNA [56], also analyzing the Alu repeats whose methylation levels are useful for evaluating the global DNA methylation. In the work performed by the Dr. Albano’s group, bone marrow samples from patients receiving azacytidine for intermediate-2/high-risk myelodysplasias were tested by ddPCR before and during treatment: as expected, a significant decrease of Alu sequences methylation after therapy compared to diagnosis was observed, so making ddPCR as an appealing instrument for DNA methylation assessment [57].

### 3.2. Digital PCR in Acute Lymphoblastic Leukemia

Acute Lymphoblastic Leukemia (ALL) is the most frequent childhood neoplasia with a dismal outcome when diagnosed in adults; in the last few years a better knowledge of B and T-ALL genetic landscape and advanced tools for MRD monitoring allowed to refine indications for alloSCT and ameliorate prognosis [58,59].

MRD monitoring in chromosome Philadelphia-positive (Ph’-positive) B-ALL is based on quantification of *BCR-ABL1* transcript using QT-PCR; a recent Italian study applied ddPCR to patients enrolled into the GIMEMA LAL2116 trial, showing optimal sensitivity (1 × 10^−5^–5 × 10^−6^) and specificity (near to 100%). In follow-up samples, ddPCR was able to reduce the proportion of positive-not-quantifiable (PNQ) cases, which represent a grey zone in the clinical practice, significantly increasing the proportion of quantifiable samples. Therefore, of the 5 cases that were negative by QT-PCR and positive by ddPCR during follow-up, 4/5 experienced a relapse, confirming the clinical relevance of a deeper MRD monitoring [60]. Similar results were found in 2018 by Dr. Coccaro and colleagues [61] and by Dr. Guan and coworkers who applied ddPCR MRD monitoring in 10 relapsed/refractory Ph’-positive ALL patients treated with anti-CD19/CD22 CAR-T-cell cocktail therapy [62]. Finally, Dr. Martinez and colleagues proposed and validated a one-step ddPCR assay for the p190 *BCR-ABL1* transcript that showed several advantages over QT-PCR: deeper sensitivity, no need for a standard curve, no need for standardization material to be shared between different laboratories for result comparison, and less propensity to the reaction inhibition [63].

In Ph’-positive neoplasms, the emergence of point mutations in Tyrosine Kinase Inhibitors (TKIs)-ligand domain of *ABL1* may represent a major barrier for success of TKIs, with T315I mutations rendering cells sensible only to the 3rd generation TKI, ponatinib. If the role of *ABL1* point mutations is well-recognized in Chronic Myeloid Leukemia (CML), in ALL the prognostic relevance of detecting small clones of T315I-mutated cells at diagnosis remains to be fully elucidated [64]. Nevertheless, in 2020 Dr. Akahoshi et al. reported that the detection of even a small amount of T315I mutation by ddPCR at the time of molecular relapse after autologous transplantation may provide appropriate information for identifying patients who are likely to develop hematological relapse [65].

About other rarer *BCR*-partner fusion genes, such as t(8;22) *BCR/FGFR1*, which confer to ALL a particularly aggressive disease course and dismal outcome, a standardized methods for MRD detection is lacking and response monitoring is usually performed with cytogenetic techniques or qualitative PCR [66]. In 2018, Dr. Coccaro et al. reported the absolute quantitation of the *BCR/FGFR1* fusion gene by a new ddPCR assay with a LOD of 0.01% [67].

In Ph’-negative ALLs (B or T), which represent most ALLs in childhood, MRD detection is more complicated and based on the Immunoglobulin *IgH, Ig, Ig* and *TCRs* rearrangement analysis; nevertheless, a consistent fraction of samples with very-low MRD levels cannot be properly quantified and must be scored as positive not quantifiable (PNQ), that represent a clinical dilemma (are they MRD positive or negative? Is it worth to proceed with transplantation or not?) [68]. Trying to address this issue, Dr. Della Starza and coworkers proposed ddPCR as an alternative method for MRD monitoring [11,69] in samples from patients enrolled in the GIMEMA LAL1308 and in the AIEOP-BFM ALL 2000 trials, finding a concordance rate of 70% between QT-PCR and ddPCR. The greater accuracy of ddPCR allowed to quantitate samples defined as PNQ by QT-PCR in a quarter of cases. To allow a better standardization, the group also proposed a fixed-threshold of positive-droplet number to define a sample as negative, PNQ or positive [69].

Another gene mutated in about 15–20% of pediatric B-ALL and in 50% of adult ALL is the *IKZF1* [70]. The presence of *IKZF1* or *BCR-ABL1* mutations has been reported to be an independent risk factor of poor prognosis [71]. In 2019, Dr. Hashiguchi and Dr. Onozawa described the application of ddPCR for detection and quantification of *IKZF1* genomic aberrations, suggesting a possible its role even as MRD marker [72].

### 3.3. Digital PCR in Lymphoproliferative Disorders (Lymphomas and Multiple Myeloma)

The *BRAF V600E* mutation interests 70–100% of hairy cell leukemias (HCL) patients [73], but also up to 50% of cases of Langerhans cell histiocytosis, especially those with skin or central nervous system involvement [74]. The clinical translation of this finding is the possibility of administering vemurafenib, already employed in melanoma [75] and in non-small lung cell cancer [76], to relapsed/refractory hematological patients carrying the *B-RAF* mutation. In a basket study, vemurafenib was administered to 26 patients with Erdheim-Chester disease or Langerhans histiocytosis: the overall response rate (ORR) was 61.5%, with long-term survival [77]. In a series of 30 relapsed/refractory HCL patients, combination of vemurafenib with rituximab offered complete response (CR) to 87% of cases, with 65% of them achieving MRD-negativity [78]. Consequently, the assessment of the *BRAFV600E* mutation is relevant both from the diagnostic and the therapeutic point of view. In 2016, our group applied an innovative ddPCR assay for detection of *BRAF* mutation to a series of 47 HCL patients: the new approach was more sensitive than QT-PCR (LOD, 5 × 10^−5^ vs. 2.5 × 10^−4^) and when ddPCR was applied as MRD marker, it was able to detect as still MRD-positive 22% of cases otherwise defined as MRD-negative by *IgH* rearrangement and 5% of cases MRD-negative by QT-PCR [79]. In 2020, a Chinese group organized an inter-laboratory quality control for the use of ddPCR for the *BRAF* mutation assessment: with a LOD of 0.02%, ddPCR was more sensitive than NGS, whose LOD was 0.3%. About the reproducibility, the 8 participants laboratories demonstrated an appropriate technical competency to perform accurate ddPCR-based measurements, with droplet volume being an important factor influencing the reaction efficiency [80].

In chronic lymphocytic leukemia (CLL), identification of *TP53* mutations, as well as of chromosome 17 deletions, that occur in about 5–10% of patients at diagnosis and more frequently at relapse, has a relevant clinical impact, making the chemo-immunotherapy not advisable for this kind of patients [81]. An Italian group proposed an interesting diagnostic workflow algorithm where a ddPCR with 6 probes for *TP53* exons 5–7 was used as first screening step. This innovative approach, with a sensitivity of 1 × 10^−3^, resulted time- and cost-effective in comparison to NGS [82]. Analogously, the chemo-immunotherapy is not the best approach for cases with *NOTCH1* mutations (that occur in approximately 10% of CLL patients at diagnosis, 20% at relapse and in over 30% of cases after Richter transformation) [83]. *NOTCH1* mutations were detected by ddPCR in 53.4% of patients, and a significant reduction of mutation load was observed after successful treatment (from a median of 11.67% to 0.09%) [84]. Subsequently, another group used ddPCR for assessing *NOTCH1* mutations in a larger series of CLL patients: with a LOD of 5 × 10^−4^, *NOTCH1* mutations were detected in 25% of the whole series and in the 55% of patients with trisomy of chromosome 12, with a significant poor prognostic impact [85].

Another field where ddPCR resulted useful for identification of patients who might benefit of Bruton Kinase inhibitors [86] is that of lymphoproliferative disorders characterized by the presence of the *MYD88L265P* mutation. This genetic abnormality triggers the anti-apoptotic *NF-kB* pathway, activates the *JAK-STAT3* and *BTK* signals, leading to the uncontrolled B cells proliferation. This mutation characterizes about 30% of Activated Diffuse Large B Cell Lymphomas (ABC-DLBCL), 52% of IgM monoclonal gammopathies (IgM-MGUS), 54% of the cutaneous DLBCL, 70% of primary DLBCL involving the central nervous system, and 90% of MW, while is absent in IgM multiple myeloma (MM) [87]. In 2018, our group contributed to set ddPCR for the identification of the MYD88 mutation; this technique detected mutation in 96% of MW and 87% of IgM-MGUS cases (vs 81% and 58% by QT-PCR, respectively); the concordance rate with QT-PCR amounted to 78% on bone marrow and 68% on peripheral blood samples; in the remaining cases, ddPCR confirmed its advantage. The most interesting finding of this work was the possible application of this molecular tool to the circulating tumor DNA (ctDNA) harvested from plasma [88] or in the cerebral spinal fluid [89]. In 2019, another group employed ddPCR for detecting *MYD88L265P* mutation in a cohort of 39 patients; with a sensitivity of 1 × 10^−3^, the authors identified the mutation in 90% of MW cases, in 44% of patients affected by LPL, in 5% of IgM MM, and no in CLL or mantle cell lymphoma (MCL) cases [90]. Another group proposed a new ddPCR assay able to detect and quantify the hot spots mutations of *EZH2, STAT6*, *MYD88*, and *CCND3* that characterize about 20% of B-cell lymphomas, especially the germinal center DLBCL (GB-DLBCL) and follicular lymphoma (FL) that seem to be associated with resistance to treatment. ddPCR, with a sensitivity of 1 × 10^−4^, was accurate either on paraffin-embedded samples or on ctDNA (the “liquid biopsy”) [91].

Because *BCL2/JH* rearrangement can be found only in 60% of FL and the assessment of *IgH* rearrangement in this lymphoma is often difficult due to its hypermutated status [92], the possibility of assessing different molecular markers is intriguing. Among them, mutations of *EZH2* are becoming relevant, even from the clinical point of view, after the recent introduction in the therapeutic armamentarium of the oral EZH2 inhibitor. In patients with relapsed/refractory FL, tazemetostat offered to EZH2-mutated patients 69% of OR and 13% of CR vs. 35% of ORR and 4% of CR to the wild-type subgroup [93]. A ddPCR for detecting *EZH2* mutations was set; interestingly, in a patient carrying two different mutations in different tumor sites, the analysis of ctDNA revealed both *EZH2* genomic aberrations, so demonstrating the optimal representativeness of liquid biopsy [94]. Even in early-stage FL, ddPCR for *BCL2/IgH* rearrangement was compared to classical QT-PCR: the concordance between the two techniques amounted to 92%, and the fusion gene was recovered by ddPCR in 18% of cases otherwise negative by QT-PCR [95].

In 2020, the European cooperative group for ddPCR published an interesting manuscript about the employ of ddPCR in 416 samples from 166 patients affected by MCL. Firstly, the authors observed a 90% of concordance rate among the 9 involved laboratories; then, they proposed some rules for performing and analyzing ddPCR reactions, such as starting from 500 ng of DNA, preferring 3 replicates, and considering as “positive” a sample showing at least 3 merged events, as “negative” that without events or with only one merged event, reserving the concept of “grey zone” (PNQ) to samples with two merged events. When ddPCR for *IgH* clonality and/or *BCL1/IgH* rearrangement was compared with QT-PCR, GeneScan PCR or flow cytometry, the respective sensitivities reached 1 × 10^−5^, 1 × 10^−4^, 5 × 10^−2^, and 1 × 10^−4^, showing once again the advantage of ddPCR in term of sensitivity [96,97].

In the T angioimmunoblastic lymphoma, the ddPCR has been proposed for the G17V mutant *RHOA* (that hyperactivates the TCR signal so prompting the abnormal T cell proliferation); with a LOD of 1 × 10^−4^, ddPCR was able to recognize mutation in 4 cases that NGS defined as unmutated [98].

In Hodgkin’s lymphoma, ddPCR was used as confirmatory tool of *STAT6* mutations on frozen biopsy tissue and ctDNA. NGS showed *STAT6* mutations in about 30% of patients, being the most frequent recurrent mutations with those of *XPO1* and *B2M*. With a sensitivity of 0.14%, ddPCR was able to recognize mutations in all cases already tested by NGS and in all cases ddPCR was able to detect mutations also on ctDNA [99].

Dr. Drandi and coworkers compared ddPCR to QT-PCR in a series of 69 patients with FL, MCL and MM: the concordance was good, and both techniques reached the LOD of 1 × 10^−5^; nevertheless, ddPCR was more accurate, because it was successful in 100% of cases, whereas QT-PCR failed in 4% of cases. This pivotal work clearly sustained the possibility of replacing ASO-PCR for *IgH* clonality with ddPCR [9].

Focusing on MM, it is incontrovertible that the prognostic value of MRD assessment is becoming a new target of treatment, thanks to the availability of drugs able to induce MRD eradication in up to 70% of patients [100]. A Japanese group recently revised the issue of MRD in autografts from 43 MM patients who underwent autologous stem cell transplantation comparing NGS (with a sensitivity of 1 × 10^−7^) to ASO-PCR (sensitivity 1 × 10^−4^/1 × 10^−5^) and to ddPCR (sensitivity 1 × 10^−5^). Correlation between ddPCR and ASO-PCR was satisfying (91%), with an advantage for ddPCR, while NGS resulted less performant [101] (Table 1).

### 3.4. Digital PCR in Chronic Myeloid Leukemia

CML is a chronic myeloproliferative neoplasm characterized by the presence of Philadelphia chromosome (Ph’) and of *BCR/ABL1* fusion gene originating from the t(9;22). TKIs (imatinib as first generation, dasatinib, nilotinib and bosutinib as second generation, ponatinib as third generation and asciminib, a new STAMP-inhibitor) are orally available drugs able to inhibit the chimeric protein function so leading to a long-term remission in more than 90% of patients [102]. Nevertheless, about one third of them must change TKI for scarce tolerability or treatment failure. In about 10% of cases, resistance to TKIs is due to point mutations in the kinase domain; among them, T315I confers resistance to all TKIs except for ponatinib and asciminib [103].

The correct management of CML patients is currently based on the serial quantitative molecular assessment of *BCR-ABL1/ABL1* ratio, which results fundamental for continuing the same TKI (in patients with optimal response), changing drug (for failing cases) or for more strictly following cases with doubt or not stable response [104]. Nevertheless, in the last 10 years a great opportunity is opened for patients with deep and stable molecular response: the attempt of TKI discontinuation (treatment-free remission or TFR), that has success in about half of cases [105]. Many efforts have been made to correctly identify patients with high probability of TFR to reduce the failure occurrence [104,106]. Among them, it is necessary to correctly identify cases in real deep molecular response (because it is known that patients in less deep response are destined to rapidly fail TFR) [107,108]. In this context, ddPCR demonstrated e good correlation with QT-PCR (99.6%), but even a superiority in terms of LOD and level of quantification (LOQ) [106,109,110,111,112]. In addition to QT-PCR, the reproducibility of results was tested on the two different commercially available platforms: the QX200 Droplet Digital PCR System and the QuantStudio 3D Digital PCR System: the concordance raised to 98.7%, with consistent results [113].

In the ISAV trial, patients with undetectable *BCR-ABL1* by ddPCR at time of TKI discontinuation more likely achieved a successful TFR. In that series, ddPCR, with a sensitivity up to 1 × 10^−7^, showed a significant negative predictive value; when ddPCR levels were combined with age, relapse rates were significantly different (100% for cases <45 years and ddPCR-positive vs 36% for patients >45 years and ddPCR-negative) [114].

Other two groups tempted to find a *BCR-ABL1/ABL1* cut off that might predict the TFR success: using ddPCR, an Italian group proposed a cut-off of 0.468 copies/mL [115], while a French group proposed 0.0023% [116]. Notwithstanding the absence of a decisive and reproducible cut off, the North American multicentric prospective “LAST” study clearly confirmed the predictive power of ddPCR: indeed, the molecular recurrence was 10% when ddPCR combined with QT-PCR confirmed the deep molecular response instead of 50% when the deep response was assessed by QT-PCR only [117]. This finding was confirmed by other authors that used ddPCR for accurately identifying patients with deep response or undetectable fusion gene at the time of TKI discontinuation [118].

In addition to the better quantitation of *BCR-ABL1* transcript, another promising use of ddPCR seems to be its use for screening *BCR-ABL1* mutations. Indeed, it has been recently established that NGS seems to be the best technique for these mutations’ identification: in the “Next in CML” study, the percentage of mutated patients increased from 25% of Sanger to 47% of NGS. Interestingly, in 69 cases NGS allowed to identify the most appropriate TKI; in 10 patients, who resulted unmutated by Sanger, NGS detected the T315I mutation, with the immediate start of ponatinib [119].

In the context of the Italian “Campus CML” working group, 44 samples were screened for T315I by Sanger, NGS and ddPCR: in our hands, the minimum mutational burden detected was 0.02%; with this sensitivity, 25 samples were concordant between ddPCR and Sanger, while 5 cases resulted mutated by ddPCR but not by Sanger. In respect of NGS, 19 samples were concordant; 2 cases, mutated by NGS, resulted wild-type by ddPCR; on the other hand, other 2 cases wild-type by NGS was mutated by ddPCR. The VAF of these cases was 0.43% and 0.39%, values under the sensitivity limit of NGS. One of the 2 failing cases in ddPCR resulted mutated on genomic DNA but not on cDNA. These data, even if preliminary, sustain the possibility of using ddPCR for a rapid screening of T315I, with the immediate therapeutic change [120].

The possibility of employing ddPCR on genomic DNA to identify quiescent leukemia stem cells is another feature distinguishing ddPCR from QT-PCR, as well shown by Dr. Albano and his group [121] and might be worth of further investigation (Table 2).

### 3.5. Digital PCR in Chronic Myeloproliferative Neoplasms

The chronic myeloproliferative neoplasms (MPNs), including essential thrombocythemia (ET), polycythemia vera (PV) and myelofibrosis (MF), are frequently characterized by the *JAK2* mutations [122,123]. Because the presence of these mutations (or, in unmutated cases, of mutations of Calreticulin (CALR) or MPL) is one of the diagnostic criteria [124], it is obvious that ddPCR was firstly set for the screening of *JAK2 V617F* mutation (that is more common than mutations at exon 12). In 2015, our group published an innovative ddPCR method for identifying and quantitating in a single reaction the *JAK2 V617F* mutation. In the 99 samples analyzed by both techniques, there was an optimal correlation between QT-PCR and ddPCR, with the latest technique showing half a log higher sensitivity than the former one (5 × 10^−4^ vs. 1 × 10^−3^). PV and MF presented a similar median mutation burden (40.45%), higher than that observed in ET (21.35%) [125], differences that we also confirmed by different grades of the spleen stiffness observed by ultrasonography [126,127,128]. Finally, a Korean group compared a ddPCR assay for *JAK2 V617F* mutation with the results from pyrosequencing, once again showing the superiority of ddPCR [129].

About CALR, it has been reported a ddPCR assay with a sensitivity of 0.01% able to quantitate the type 1 mutation; even in this case, ddPCR was predictive of the clinical outcome [130].

**Table 2:** ddPCR and myeloid disorders.

Disease	Target	Reference
**AML**	**NPM1** **IDH1/IDH2** **WT1** **PML-RARa** ***PML-A216V*** **C-kit**	[32,33,34,39,41,42,48,51,52,55]
**CML**	**BCR-ABL1** **T315I**	[108,109,110,111,112,113,119]
**MDS**	**Alu methylation**	[57]
**MPNs**	**JAK2** **CALR**	[123,124,127,129]

### 3.6. Digital PCR in Transplant and Immunoterapies

Allogeneic hematopoietic stem cell transplantation (AlloSCT) is a potentially curative therapeutic option for several high-risk hematological malignancies (AML, ALL, MDS, lymphomas), especially if performed in CR. After AlloSCT the follow-up is principally based on chimerism and, when possible, on disease specific MRD markers or persistence of mutations: all these strategies allow to promptly detect and treat graft rejection or disease relapse [131]. Nevertheless, the correct timing, samples source—PB or BM—and techniques for chimerism evaluation as well as the exact threshold to distinguish complete donor chimerism from mixed chimerism are still matters of debate [132,133,134].

Currently, the standard methods to measure chimerism are QT-PCR-based analysis of Short Tandem Repeats (STR), with a sensitivity between 5% and 1%, according to the diversity of donor/recipient fingerprint [135]. During the last few years, several studies tried to apply ddPCR to the chimerism assessment, even for levels <1% [136,137]. One of the proposed strategies for children who underwent transplantation for primary immunodeficiency diseases included ddPCR for *SRY* and *RPP30* genes that allowed detect the male/female chimerism. This method revealed accurate and was able to analyze very small amount of genomic material (less than 10 ng) [137]. With a sensitivity of 8 × 10^−5^, the correlation between STR and ddPCR was higher than 99%, thus supporting the use of ddPCR also for the chimerism assessment [138].

In AlloSCT for malignancies when a suitable MRD marker is available, the better clinical management could be obtained by integrating chimerism analysis with MRD monitoring; Dr. Waterhouse et al. reported a combined use of ddPCR for chimerism and MRD in a series of 70 patients who underwent transplantation, mainly for myeloid malignancies. The authors reported a high concordance between mixed chimerism detection and MRD values, when *NPM1, DNMT3A, MLL-PTD, IDH1* and *KRAS* were monitored [139].

In line with these results, a Japanese group assessed by ddPCR (sensitivity 1 × 10^−5^) the presence of T315I mutation in 25 patients with Ph’-positive ALL who underwent alloSCT. The hematological relapse was predicted by the persistence/reapperance of the mutation after alloSCT, even at sub-clonal levels (median ratio T315I/ABL1 = 0.91%) [65].

Finally, a ddPCR assay was set for evaluating the immune reconstitution in MM patients after autologous transplantation. Indeed, during TCR rearrangement, excised DNA fragments create the TCR excision circles (TRECs) that have no clear functions but can be used for determining the thymus activity and output [140]. Our group used a new ddPCR for measuring TRECs in 9 patients with MM who underwent autologous transplantation and received high-dose zinc supplementation versus other 9 that did not receive zinc. Interestingly, zinc supplementation supported the immune reconstitution: indeed, TRECs significantly increased from day +30 until day +100 only in the zinc group (6.1-fold vs 1.8 in the control group) [141].

Another innovative field of ddPCR application is represented by the immunotherapy, and in particular by the administration of CAR-T cells to patients affected by CD19+ relapsed/refractory DLBCL, MCL or ALL [142]. Indeed, the shorter or longer persistence of CAR-T seems to be predictive of success [143], whereas it is not still clear the impact of CAR-T persistence on adverse events, such as the cytokines release syndrome (CRS) or immune effector cell-associated neurotoxicity syndrome (ICANS) [144]. In 2020, two different German groups developed new ddPCR assays for monitoring patients receiving CAR-T. In the first work, published by Dr. Fehse and coworkers, starting from 120 ng of DNA the authors reached a sensitivity of 0.01%. Interestingly, the CAR-T expansion above the median peak level of 11.2/mL was correlated with better clinical responses, whereas treatment was less effective in patients for whom CAR-T peaks were below the median [145].

In the paper by Dr. Mika et al. detection and quantification of CAR-T were feasible in all patients, once again with a sensitivity of 1 × 10^−4^. As expected, significant differences in CAR-T expansion were observed: in 4 patients the initial CAR-T expansion was followed by decreasing numbers of copies; in the other 3, CAR-T were still detectable after 9 months from infusion and the CAR-T persistence and expansion were associated with better clinical responses; in this series, higher levels of CAR-T correlated also with ICANS but not with CRS occurrence [146].

A third group added to the ddPCR for quantitating CAR-T a ddPCR assay for measuring *IL-6* gene expression. Differently from that expected, *IL-6* gene levels were not predictive for the development of CRS but might be useful for triggering tocilizumab treatment at the first clinical signs of CRS. From the CAR-T expansion and clearance point of view, 4 different patterns have been described by these authors: that of rapid increase and rapid decrease with complete disappearance of CAR-T, that rapid increase and slow decrease with higher persistence, that of rapid increase and rapid decrease with lower persistence, and that of slow increase but rapid decrease with almost disappearance. Interestingly, patients assigned to the category “rapid increase and slow decrease with higher persistence” seemed to have the best response rate, but also a higher risk of CRS, independently from the *IL-6* gene expression [147] (Table 3).

## 4. Conclusions

Born about 20 years ago, ddPCR is a new version of QT-PCR, more sensitive, specific, and accurate. With a LOD ranging from 10^−4^ and 10^−5^ according to different assays, ddPCR allows to quantitate about one quarter of samples already defined as PNQ by QT-PCR, so making more easily the patients’ management and follow-up. The versatility of this technique makes it available for measuring gene expression (without the need of a standard curve or plasmids), but also for detecting single or multiple point mutations, either on cDNA but also on genomic DNA, both on bone marrow, peripheral blood or liquid biopsy.

As above reported, many are the hematological contexts where ddPCR has been used and implemented: acute leukemias, where it is able to quantitate *NPM1* mutations but also *WT1* expression; Ph’-positive leukemias, where it is used for measuring more accurately the *BCR-ABL1/ABL1* ratio to also identify the patients best candidate to TFR but also for *BCR-ABL1* mutations detection; the MPNs, where *JAK2* and *CALR* mutations have a clear diagnostic role, and the lymphoma/myeloma setting, where *IgH* and *TCR* clonality can be combined with *BCL2/JH* and *BCL1/JH* fusion genes for assessing MRD. Finally, ddPCR can be used for chimerism determination and for monitoring immune reconstitution and CAR-T persistence in patients who receive transplantation or the new immunotherapies (Table 4).

In respect of NGS, ddPCR is more sensitive (at least 2 logs), but also more easily optimizable and fast in producing results. Indeed, we must consider that for reducing NGS costs more than one sample must be loaded into the cartridge, with increased time for results availability. On the other hand, we must keep in mind that ddPCR is not able to recognize all possible nucleotide changes, and that is appliable essentially to few known hot spot mutations. Nevertheless, as in the case of T315I, a rapid identification of a mutation that might induce a rapid therapeutic change might be worth of consideration.

Probably, the best diagnostic algorithm must put together all available sensitive and specific molecular techniques (QT-PCR, ddPCR, NGS) to help physician to do a “reasoned” therapeutic or follow-up approach that would lead to reach the modern goal of hematologists: the “patient-tailored” therapy. In this scenario, we must consider ddPCR as a good allied.

## Figures and Tables

**Table 1:** ddPCR and lymphoproliferative neoplasms.

Disease	Target	Reference
**ALL**	**IgH** **BCR-ABL1**	[60,61,62,63,65]
**HCL**	**B-RAF**	[79,80]
**CLL**	**TP53** **NOTCH1**	[82,84,85]
**WM**	**MYD88**	[88,89,90]
**HL**	**STAT6**	[98]
**FL**	**EZH2** **BCL2/JH**	[94,95]
**MCL**	**BCL1/JH**	[96]
**MM**	**IgH**	[99,100]

**Table 3:** ddPCR and immunotherapies.

Disease	Target	Reference
**ALLOGENEIC TRANSPLANTATION**	**Chimerism** **Chimerism & MRD** **T315I**	[133,135,136,137,138,139]
**AUTOLOGOUS TRANSPLANTATION**	**TRECs**	[141]
**IMMUNOTHERAPY**	**CAR-T**	[145,146,147]

**Table 4:** Comparison among different quantitative molecular techniques.

Technique	Target	Quantitation	Sensitivity	Advantages	Disvantages
**QT-PCR**	Gene expression Mutations (hot spot) Recurrent fusion genes At diagnosis MRD	Relative (in comparison to itself in a previous time-point, using reference curve)	10^−4^/10^−5^	Fast, widely applicable, sensitive, specific, accurate, standardized. Applicable even to a single sample	No adapt for clonality in case of clonal evolution High risk of contamination
**NGS**	Mutations (whole gene) Better at diagnosis than as MRD tool	Absolute	1% For clonality assays, 10^−4^	Able to detect any mutation Good tool for clonality (unsupervised vision), not influenced by clonal evolution Low risk of contamination	Not widely applicable No standardized Time-consuming Higher costs Applicable to a right number of samples
**ddPCR**	Gene expression Mutations (hot spots) Recurrent fusion genes At diagnosis MRD	Absolute	10^−4^/10^−5^	Fast, sensitive, specific, accurate, standardized. Applicable even to a single sample Low risk of contamination	Not widely applicable No standardized No adapt for clonality in case of clonal evolution

## Data Availability

The data are not publicly available due to privacy.
